# “Back to the future” projections for COVID-19 surges

**DOI:** 10.1371/journal.pone.0296964

**Published:** 2024-01-30

**Authors:** J. Sunil Rao, Tianhao Liu, Daniel Andrés Díaz-Pachón

**Affiliations:** 1 Division of Biostatistics, University of Minnesota, Minneapolis, Minnesota, United States of America; 2 Division of Biostatistics, University of Miami, Miami, Florida, United States of America; University of Galway, Ireland / Anhui University of Finance and Economics, CHINA

## Abstract

We argue that information from countries who had earlier COVID-19 surges can be used to inform another country’s current model, then generating what we call *back-to-the-future* (BTF) projections. We show that these projections can be used to accurately predict future COVID-19 surges *prior to an inflection point of the daily infection curve*. We show, across 12 different countries from all populated continents around the world, that our method can often predict future surges in scenarios where the traditional approaches would always predict no future surges. However, as expected, BTF projections cannot accurately predict a surge due to the emergence of a new variant. To generate BTF projections, we make use of a matching scheme for asynchronous time series combined with a response coaching SIR model.

## Introduction

“The past is prologue” (Shakespeare). “The best predictor of future behavior is past behavior” (Twain). “The best way to predict the future is to study the past or prognosticate” (Kiyosaki). These are all famous quotes which, when applied to important prediction or projection problems (projection being prediction into the future), suggest that a careful understanding of past events is essential to predicting future trends.

Analyzing *past* estimators of prevalence is somewhat more standard [1–3]. However, forecasting is a more daunting task. For instance, when applied to the problem of projecting a new surge of COVID-19 infections in India, back in mid February 2021, known forecasting strategies did not work. India had seen a remarkable downturn in their daily new cases curve and all models built at that time were projecting a continuing trend in that direction, down towards zero daily new cases. All sorts of explanations were produced to why India escaped relatively unscathed, including cross-protection from other regular vaccines, like the BCG TB vaccine; a younger age distribution to the population; a warmer climate; and more homes with open window settings [4].

But by late March or early April, a significant upturn in the daily new cases curve had taken hold and India was rapidly experiencing a second surge dramatically more ferocious than the first. In fact, daily new cases counts would cross the 400K per day soon thereafter (reported cases granted and likely hugely under-counted), with a lagging rise in the number of hospitalizations and deaths.

So if modeling using the first surge data was not informative, was there any way to objectively predict the second surge? And to make things even more challenging, can a future surge be predicted before that surge has actually started? That is, prior to the inflection point between the ending of a current surge and the start of a new one. We argue surprisingly that there may be. In this paper we present a method called *back-to-the-future* (BTF) projections that borrows information from so-called “matching” countries that experienced an earlier surge. This information is used to coach projections forward in time. In fact, when applied to India, BTF, in spite of the fact that it cannot forecast the surge of the new delta variant because there was no previous surge to compare against, it explains very well the surge of previously existing variants of COVID-19.

This paper is organized as follows. We begin with a short review of the basic modeling strategies for pandemic data and why projections are so sensitive to the point of inflection. We then introduce the BTF idea and algorithm for fitting. Empirical results on 12 different countries from every continent except Antarctica are presented with comparisons against the basic modeling approaches and competitors. We finally provide some justification for the matching and coaching used in making BTF projections.

## Contrasting modeling strategies for pandemics

### Compartment models

The SIR model is the simplest compartment model for describing the evolving dynamics of an epidemic through a population. It can be described by a set of ordinary differential equations (ODEs),

$$\begin{array}{c} \begin{array}{cc} \frac{dS}{dt} & =-\frac{\beta I(t)S(t)}{N},\\ \frac{dI}{dt} & =\frac{\beta I(t)S(t)}{N}-\gamma I\left(t\right),\\ \frac{dR}{dt} & =\gamma I(t), \end{array} \end{array}$$ (1)

where, at time *t*, *S* is the susceptible population, *I* is the number of infectious, *R* is the number removed either by death or recovery, and *N* is the sum of these three:

$$\begin{array}{c} S(t)+I(t)+R(t)=N. \end{array}$$

The parameters *β* and *γ* are the transmission and recovery rates, respectively. From Eq (1),

$$\begin{array}{c} \frac{dS}{dt}+\frac{dI}{dt}+\frac{dR}{dt}=0. \end{array}$$

Also from Eq (1), dividing the first equation by the third, and integrating with respect to *S* and *R*,

$$\begin{array}{c} S\left(t\right)=S\left(0\right)e^{-R_{0}\left(R\left(t\right)-R\left(0\right)\right)/N}, \end{array}$$

where *R*_0_ is the basic reproduction number given by *R*_0_ = *β*/*γ*. At the outset of an epidemic, when *S* ≈ *N*, infection numbers begin to surge as *R*_0_ ≫ 1. Subsequent surges are characterized by the ratio *N*/*S*. When *R*_0_ > *N*/*S*, infection numbers rise more rapidly, hit a peak when *R*_0_ = *N*/*S*, and then decline as *R*_0_ < *N*/*S*.

When assumed purely mechanistic, numerical methods such as Euler discretization or the Runge-Kutta approximation method [5] can be used to obtain approximate solutions of the ODEs with given boundary conditions. In a statistical analysis framework, a model is constructed with a deterministic and random component. The former is the SIR model itself. The latter allows for a random sampling scheme, thus creating a stochastic extension of the mechanistic model. Parameter estimation can be done via frequentist optimization, like least squares, the method of moments, maximum likelihood estimation, or Bayesian approaches using Markov Chain Monte Carlo techniques. A clear advantage of the stochastic extensions is the ability to quantify uncertainty in parameter estimation and prediction due to sampling variability. A full account of the SIR model (and related compartment model extensions) can be found in [6].

### Time series ARIMA models

Time series models have also been exploited for modeling epidemic data trends [7, 8]. Using new notation, we will let the daily infection counts be *Y*_*t*_ and defined Δ^*d*^*Y*_*t*_ = (1 − *L*)^*d*^*Y*_*t*_ = *Y*_*t*_ − *Y*_*t* − *d*_, where *d* is the number of differences needed to make the series stationary, then the ARIMA(*p*, *d*, *q*) model (Box and Jenkins 1976) has the form

$$\begin{array}{c} A\left(L\right){\left(1-L\right)}^{d}Y_{t}=\delta +\Omega \left(L\right)\epsilon_{t}, \end{array}$$ (2)

where *A*(*L*) = 1 − *α*_1_*L* − … − *α*_*p*_*L*^*p*^, Ω(*L*) = 1 − *θ*_1_*L* − … − *θ*_*q*_*L*^*q*^, *p* is the autoregressive order, *q* the moving average order, and *L* is known as the backshift operator. The random variable *ϵ*_*t*_ is white noise assumed to follow a normal distribution. The *α*_1_, …, *α*_*p*_ are the autoregressive parameters, and the *θ*_1_, …, *θ*_*q*_ are the moving average parameters, both sets to be estimated by maximum likelihood. The order of the ARIMA(*p*, *d*, *q*) model is typically chosen using a model selection criterion, like BIC [9] or AIC [10], among other methods.

### Curve fitting

Curve fitting essentially amounts to deriving a functional relationship between *Y*_*t*_ and *t* such that the estimated curve matches the observed daily infection count trend as closely as possible. This approach is generally considered less tied to underlying assumptions about features within the population that might be driving the daily infection numbers. However, the drawback is that it’s not a mechanistic approach and thus may not do as well with longer term forecasts. Some examples include the generalized logistic model [11] and the generalized Gaussian cdf [12], both adopted by the Institute of Heath Metrics and Evaluation (IHME). These models have been extended to allow for incorporation of covariates that can connect different locations together (https://ihmeuw-msca.github.io/CurveFit/methods/).

### Error correction model (ECM) for short term forecasts

In [13], the authors developed a method for short term COVID-19 forecasting called error correction model (ECM), that has some similarities to the method we will describe shortly. ECM uses a lasso-based [14] approach to connect a country of interest at a point in time to other countries that experienced an earlier surge. The idea was to adjust the short-run dynamics to a departures from long-run relationships between the country of interest and the other countries experiencing earlier surges. Tracking of dynamics of the other countries then permits short term forecasting for the country of interest. The model can be expressed as

$$\begin{array}{c} \Delta y_{t}=\Delta x_{t}^{\prime }\alpha +\gamma \left(y_{t-1}-x_{t-1}^{\prime }\beta \right)+\varepsilon_{t}, \end{array}$$ (3)

where Δ, *γ* and *β* are unknown parameters and *ε*_*t*_ is a mean zero, fixed variance random noise. Also, *y* corresponds to the country of interest, *t* is the point ahead in time after *c* case counts, *x* is the vector of observed case counts at time *t* for other countries that reached *c* case counts earlier than the country of interest.

A two-step estimation algorithm was proposed by the authors: in the first step, *β* was estimated by $\hat{\beta }$ using the lasso; for the second step, *α* and *γ* were estimated by ordinary least squares estimation, given the first-step value $\hat{\beta }$.

A rolling window approach where the model is continually updated is advocated in order to adjust to the dynamic nature of the pandemic. The authors demonstrated empirically over 14 prediction horizons that their methodology produced smaller mean absolute percentage errors (MAPEs) than a simple quadratic trend regression approach and an integrated AR model of order 1. They examined both the prediction of future cases and deaths. In section, we define conditional absolute errors and show that BTF outperforms ECM for the data analyzed in the present paper.

### Surge prediction and the sensitivity to point of inflection

The focus in this paper is to predict future COVID-19 surges *before* an inflection point for the surge itself—in other words, on the downward trajectory of the previous surge or in a valley before the future surge.

**Fig 1:**
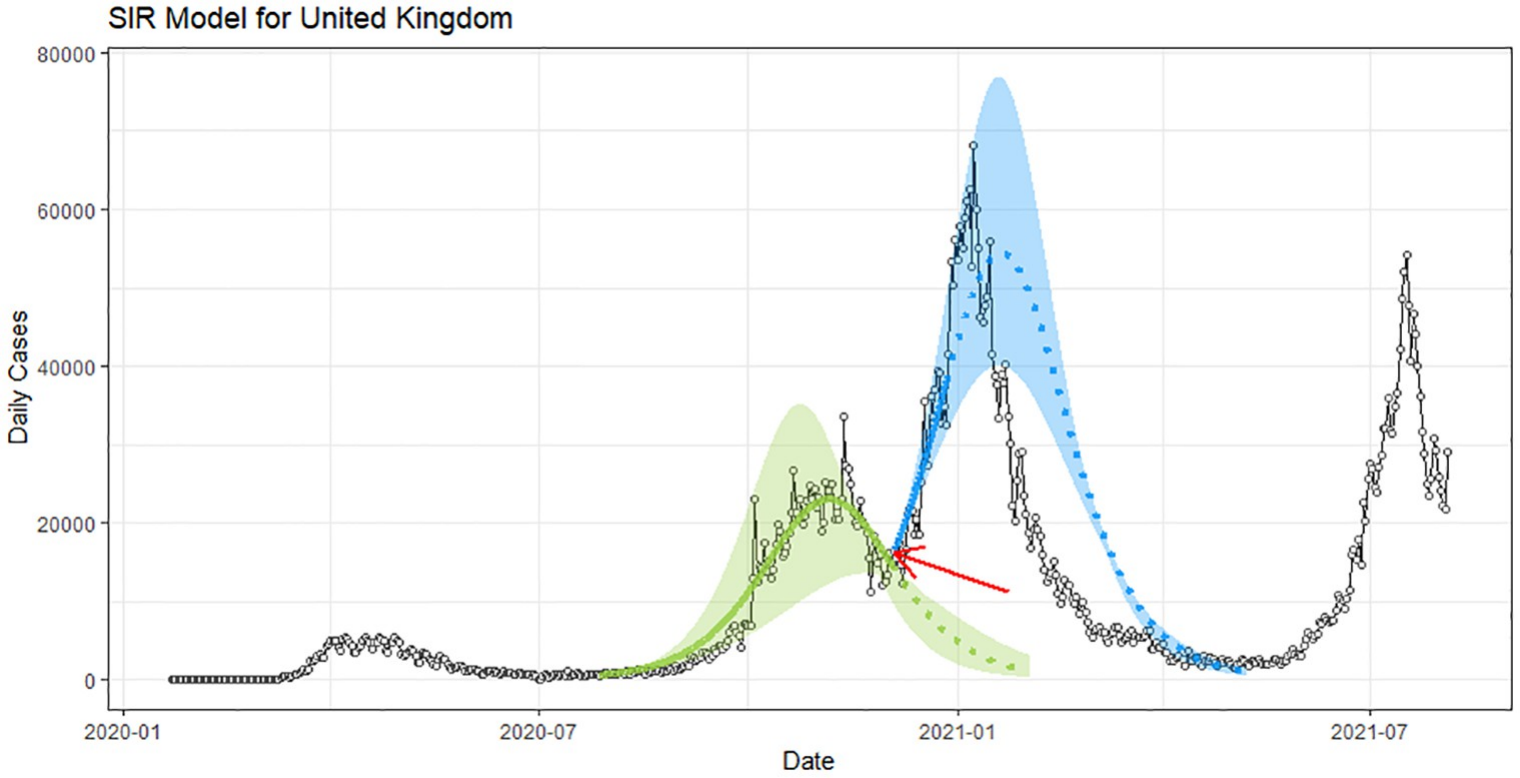
Sensitivity of the UK SIR model projections to the inflection point (red arrow) of daily infections curve.

Projections before and after an inflection point can be markedly different. To illustrate this point, consider SIR models fit to daily case count data for the United Kingdom, as shown in Fig 1. The red arrow on the plot indicates a point of inflection before the start of the third surge around November, 2021. Let’s suppose this is the surge we are trying to predict. The green curve is an SIR model fit to data prior to the inflection point that is been projected forward past the inflection point in Fig 1. Notice how it’s descending to zero. Now assume we wait some days to make the projection for the third surge. The projected curve from such an SIR model would look like the blue curve in the figure. As expected, it is rising upwards towards the observed peak daily count. This looks to be much more accurate. These types of projections are of less public health planning value, since with highly contagious viruses like the omicron variant of COVID-19, with an *R*_0_ number estimated to be near 10, it is nearly impossible to blunt the surge after the point of inflection because one is always “running behind” the virus. Our proposed methodology seeks to do better than the green curve based on the same observed data.

## Back to the future projections

We now restrict our attention to one particular sequence—the daily infection counts over time. Our main interest is to project a future oncoming next surge during the downward trajectory of the current surge *but prior to an inflection point in the curve that might indicate the start of a new surge*. As just shown, standard approaches will have all projected curves descending down towards zero daily counts.

To improve naive projections, we exploit the very nature of a pandemic—the fact that infections are spreading asynchronously in time across different countries. Countries (*B*_*m*_, *m* = 1, …, *M*) that have experienced a surge earlier in time may provide useful information in making projections forward in time for a country of interest (*A*). This is done by estimating an ARIMA time series model for *A* for the current surge *S*_1_ (say ${\hat{f}}_{t\in S_{1}}\left(A\right)$), shifting this curve backwards in time and overlaying its fitted curve with fitted curves from the other *B*_*m*_ countries *previous* surges (*S*_1,*m*_) (say ${\hat{f}}_{t\in S_{1,m}}\left(B_{m}\right)$). A determination of best country match is then made based upon the pairwise difference in fitted curves ${\hat{f}}_{t\in S_{1}}\left(A\right)$ and ${\hat{f}}_{t\in S_{1,m}}\left(B_{m}\right)$.

Once the best match (${\tilde{B}}_{m}$) amongst the *M* countries has been established, an SIR model is fit to the *observed* daily infection counts forward in time for ${\tilde{B}}_{m}$. *This data is actually observed*, which is a key fact. Relevant SIR curve parameter estimates are then passed to country *A*, using *A*’s current initial conditions, to make *not yet observed* projections forward in time. This is a type of statistical *coaching*. It’s useful to impose a short “washout” period to allow the current surge to come to completion. We call this *Back to the Future* (BTF) projections. The steps can be summarized in the algorithm:

**Back to the Future Projection Algorithm**

Suppose we want to project country *A* after *A*’s *i*-th surge.

1. Select candidate countries such that
  - its *i*-th surge happens before *A*’s *i*-th surge,
  - it has sufficient data for *i*-th surge to match with *A*’s *i*-th surge (same length interval),
  Denote these candidate countries as ${\left\{B_{k}\right\}}_{k=1}^{K}$.
2. Fit ARIMA models for the *i*-th surge of *A* and *B*_*k*_.
  Smooth these fits using cubic smoothing splines, with the degree of smoothing determined by leave-one-out cross-validation.
  Denote the fitted models by $\hat{A}$ and ${\hat{B}}_{k}$.
3. From ${\left\{B_{k}\right\}}_{k=1}^{K}$, select the country most similar to *A* by
  $$\begin{array}{c} {\tilde{B}}_{m}\equiv \text{arg}\;{\text{min}}_{B_{k}}\{\text{median}[{\hat{A}}^{sd}-{\hat{B}}_{k}^{sd}]\}, \end{array}$$
  where ${\hat{A}}^{sd},{\hat{B}}_{k}^{sd}$ be the standardization of $\hat{A},{\hat{B}}_{k}$ by its maximum.
4. Fit an SIR model to ${\tilde{B}}_{m}$ after its *i*-th surge (a 10-day gap may be introduced to washout the effect of the *i*-th surge).
5. Pass the estimated parameters $\hat{\beta }\left({\tilde{B}}_{m}\right),\hat{\gamma }\left({\tilde{B}}_{m}\right)$ by the SIR model of ${\tilde{B}}_{m}$ to the SIR model with *A*’s initial conditions.
6. Generate the projection using this new SIR model.

### Sensitivity analysis

To analyze the sensitivity of the SIR model, we jitter the two parameters *β* and *γ* for a small amount *δ*. In practice, we sample the parameter pair by a uniform distribution on [*β* − *δ*, *β* + *δ*] × [*γ* − *δ*, *γ* + *δ*] (in our cases *δ* is chosen as 0.01). Then run the SIR model by each pair of these parameters. We can shade the union of these individual runs.

### Why coaching using a compartment model?

The mechanistic nature of the systematic component of the compartment model provides a more parsimonious representation of country ${\tilde{B}}_{m}$ over a longer period of time. It also allows a clear path to incorporating country *A*’s specific characteristics. This helps to anchor the BTF projections and to generate more accurate projected trends over longer windows of time, rather than purely generating accurate short-term projections which are of limited public health benefit.

Contrast this to coaching using an ARIMA model instead from country ${\tilde{B}}_{m}$. Since only lagged effects can be modeled in the ARIMA model, country ${\tilde{B}}_{m}$’s shape of their next surge (after the matching one), will not fully inform country *A* forecasts of interest.

## Data

Killeen *et al.* [15] assembled the dataset under focus. COVID-19 infection volume time series came from the Johns Hopkins University CSSE COVID-19 Tracking Project and Dashboard(https://coronavirus.jhu.edu/data) for which data was pulled from the time range 01/22/2020 until 09/18/2021.

## Performance on a selection of countries

A BTF analysis was carried out for a selection of 12 countries from all 6 populated continents around the world. Thus the performance of our methodology was examined regardless of the regional variation that might exist from continent to continent. In particular, the chosen countries experienced second surges during our time window of analysis and the goal was to accurately forecast second surges from a lagged time point towards the end of their first surges (i.e. before the inflection point of the daily infection curve happened, indicating the start of a potential second surge). This would be a truly honest projection and would more clearly demonstrate the utility of the BTF methodology. Usual forecasting with compartment models, ARIMA models, or curve fitting, would all indicate the projected curves continue downwards, given that the projections were made from a point in time on the downward trajectory of the first surges. As a negative control, we also included Australia where no second surge was detected during the analysis time window.

**Fig 2:**
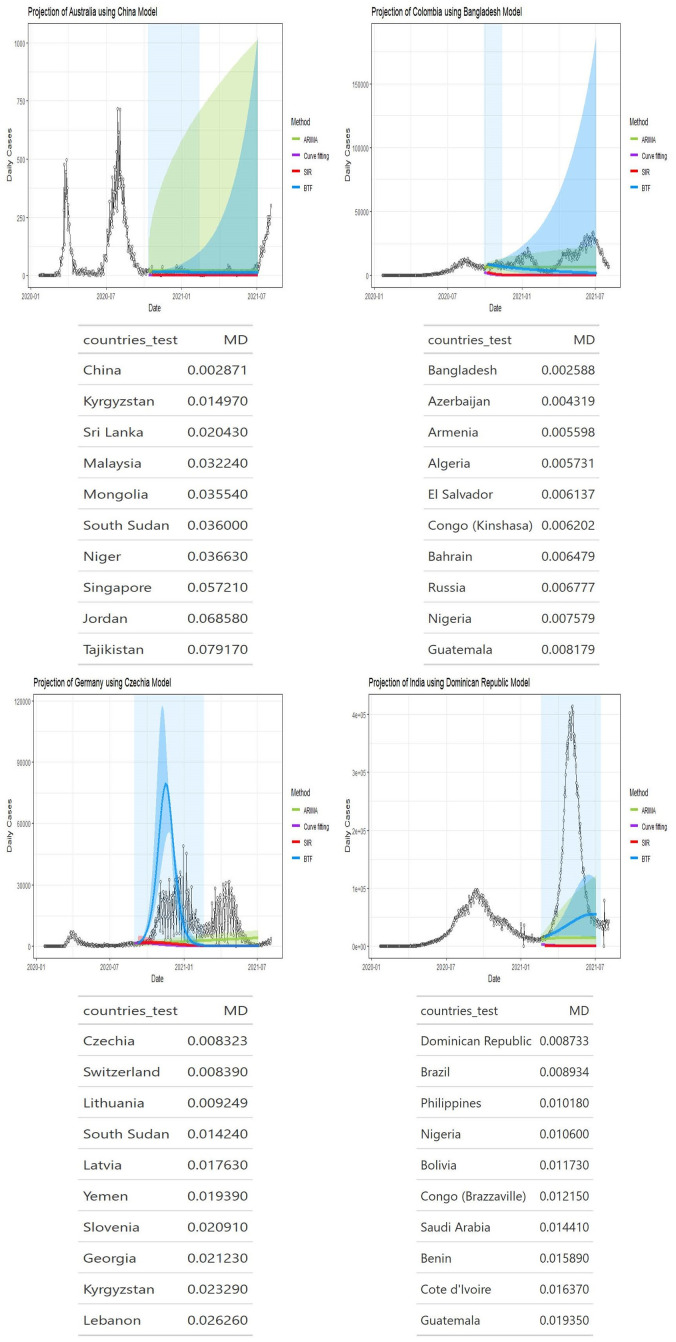
Projection curves for Australia, Colombia, Germany, and India, using BFT projections. Projected blue curve and region of projection (before inflection point) of next surge in shaded blue. Note that a 10 day washout period is forced before projections start. Matching country ranking tables shown underneath each plot.

**Fig 3:**
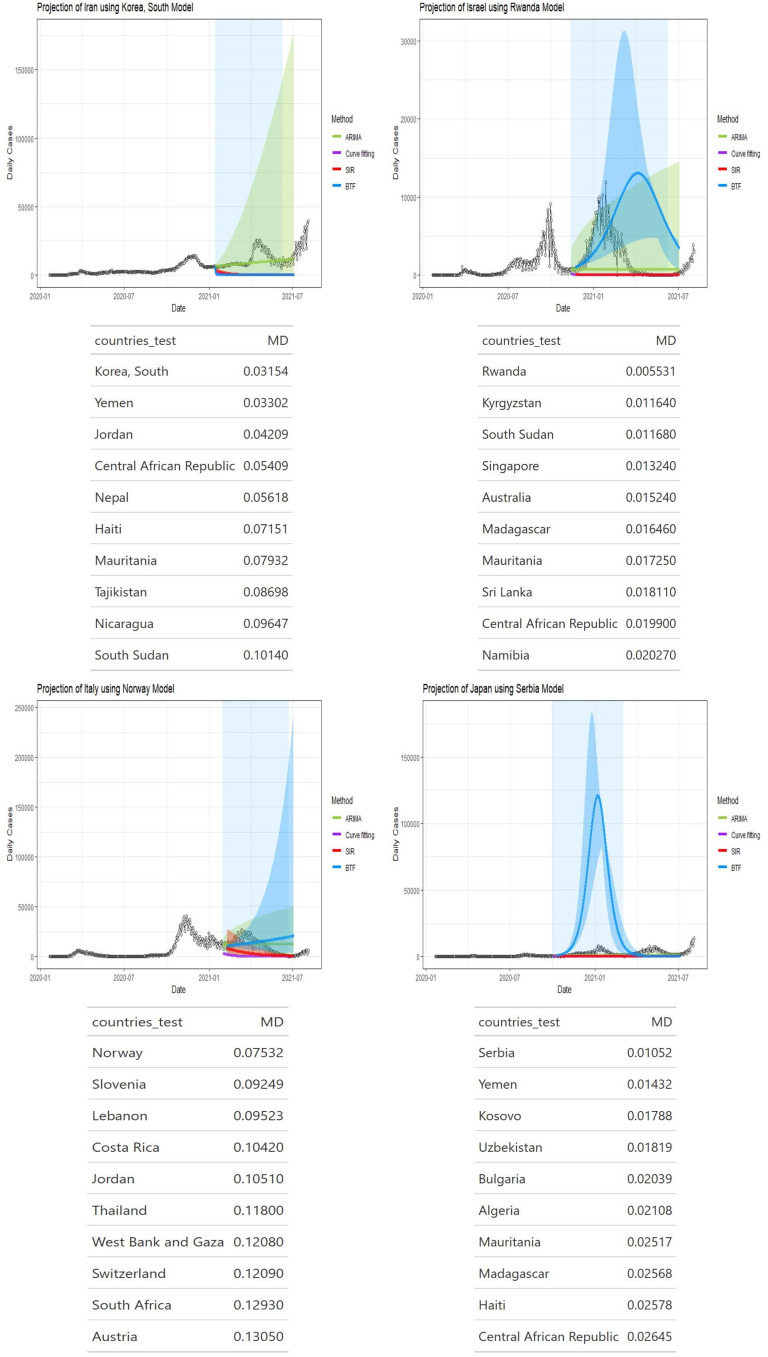
Projection curves and matching country ranking tables for Iran, Israel, Italy, and Japan, using BFT projections.

**Fig 4:**
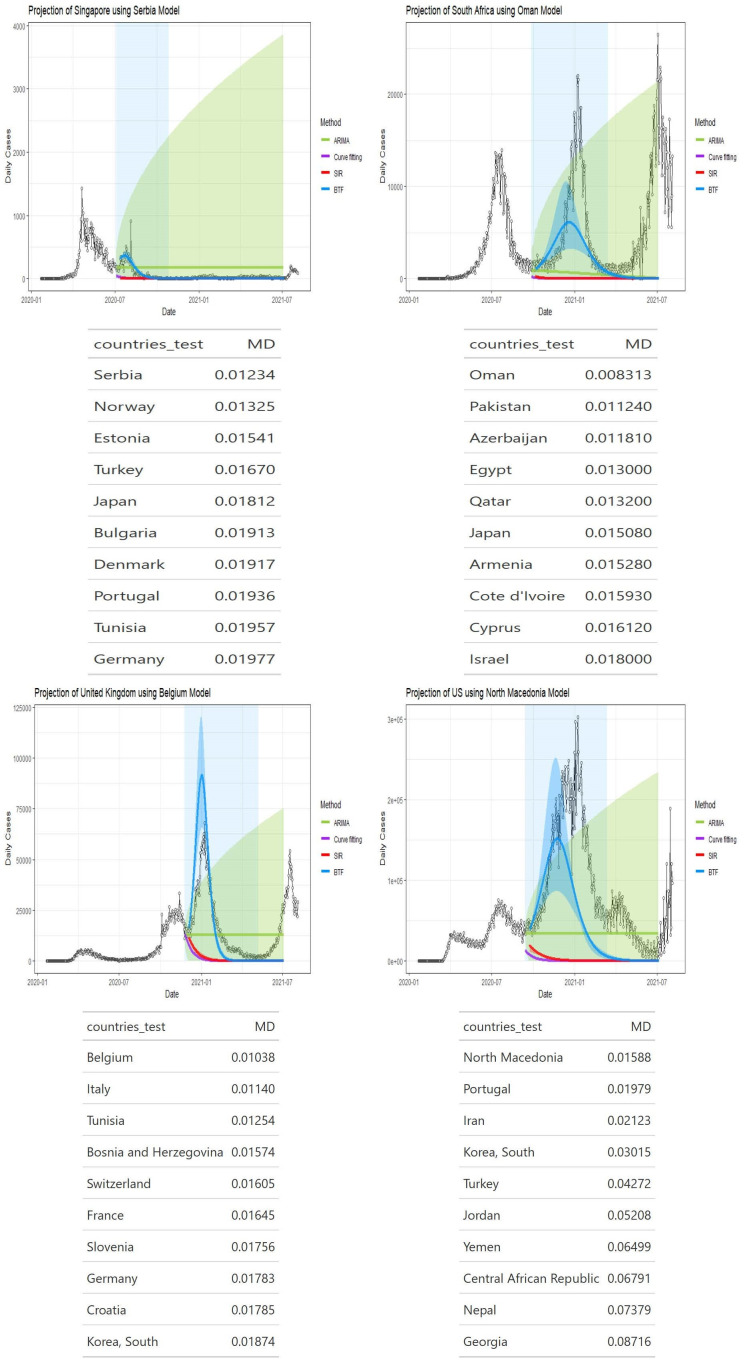
Projection curves and matching country ranking tables for Singapore, South Africa, United Kingdom, and United States, using BFT projections.

Figs 2–4 show four panels each with each panel depicting the following: i) a observed daily infection curve; ii) BTF projected curves (solid blue curve) with sensitivity bands (darker blue shaded); iii) standard SIR projected curved (red) with sensitivity band (red shaded); iv) standard ARIMA(*p*, *d*, *q*) forecast (green curve) with 95% prediction interval (green shaded) and v) generalized logistic growth curve model with 95% bootstrap prediction intervals (purple curve and shaded regions). The time window of each surge of interest are the blue rectangular regions. Underneath each country’s plot is the matching table from which the coaching country’s curve was derived.

Making these kinds of projections is clearly a very challenging task and represent a type of aspirational goal (see [16]). Thus, judging the accuracy of the BTF projections must be calibrated appropriately. For point estimate-based predictions, one can use absolute error; for interval-based predictions, the weighted integrated score is an option [16].

**Fig 5:**
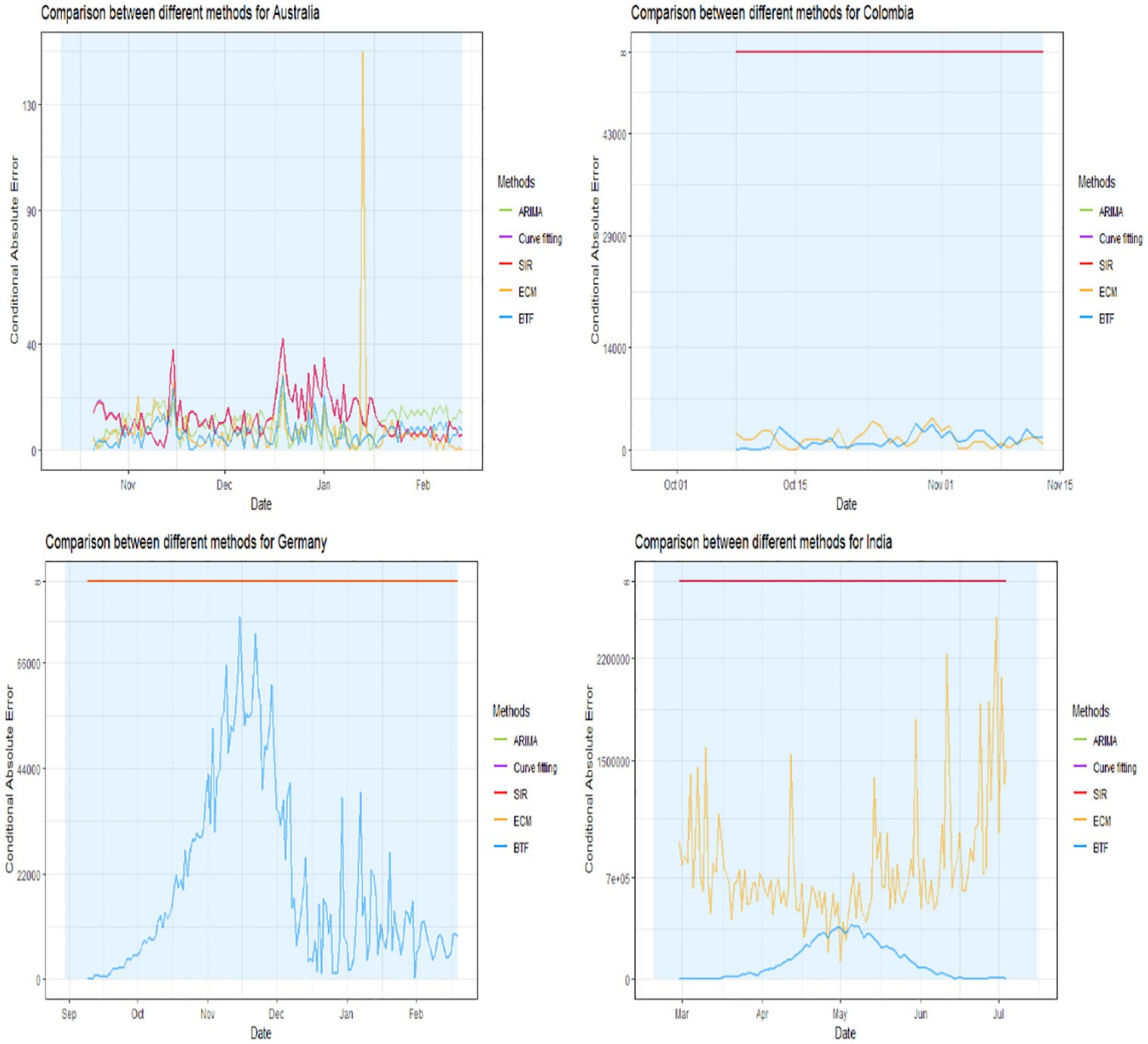
CAE estimates for Australia, Colombia, Germany, and India.

**Fig 6:**
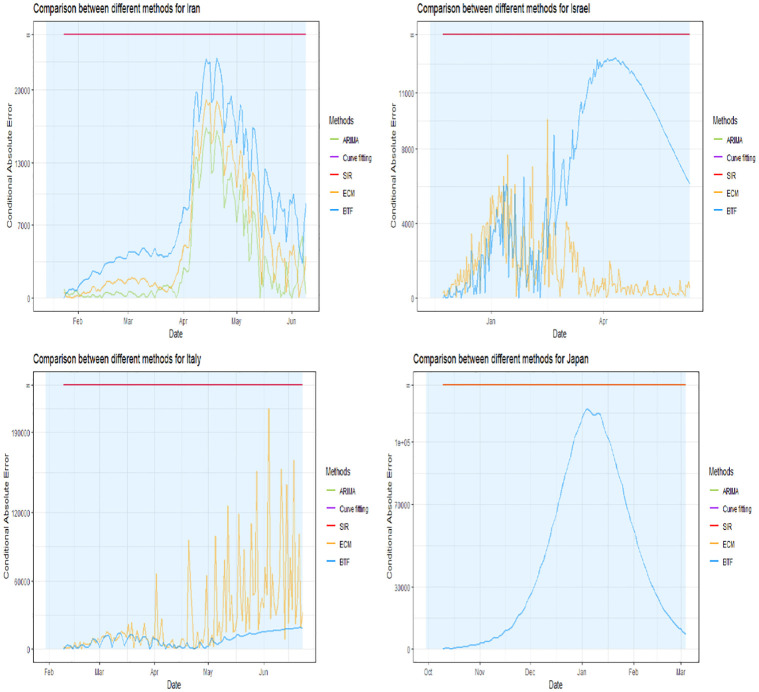
CAE estimates for Iran, Israel, Italy, and Japan.

**Fig 7:**
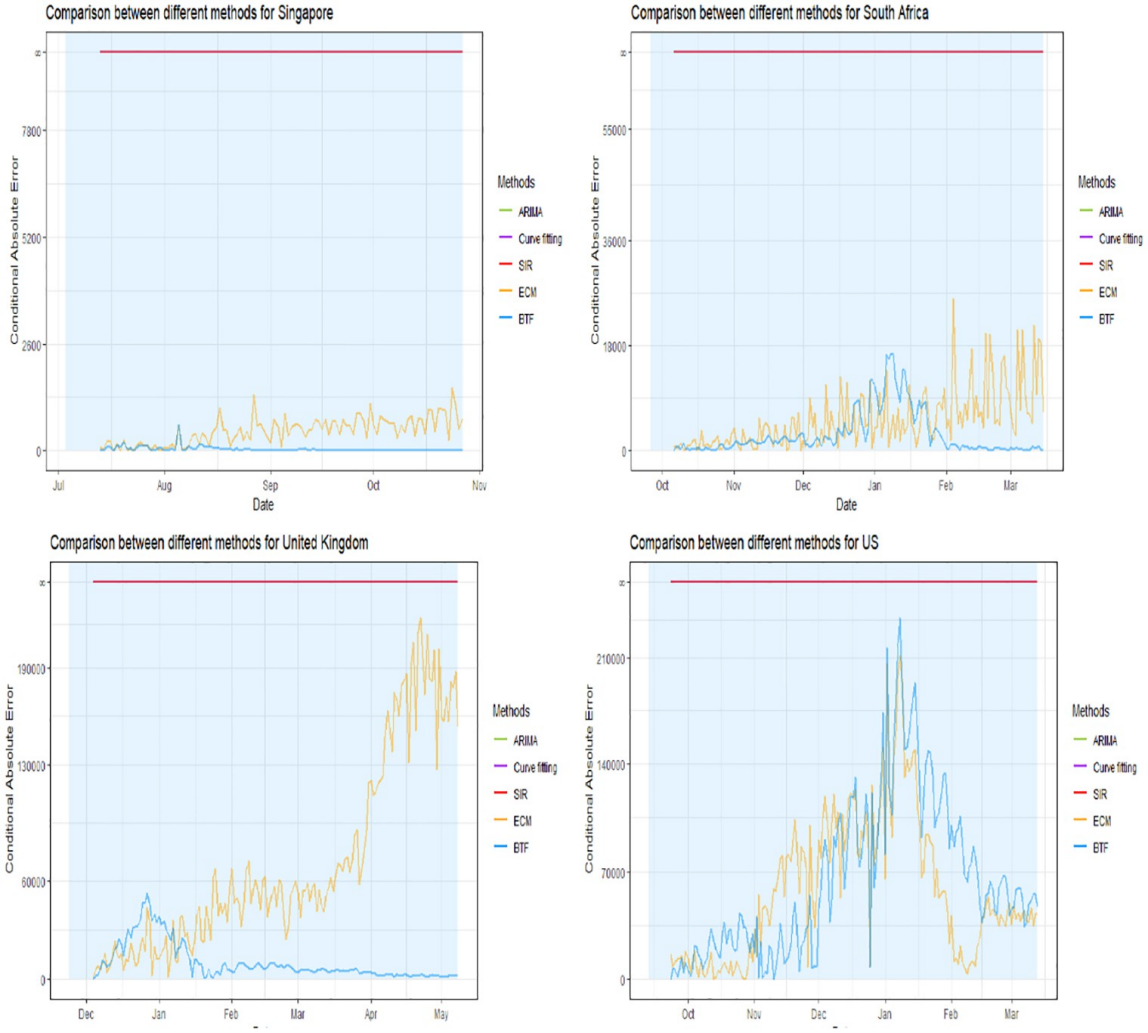
CAE estimates for Singapore, South Africa, United Kingdom, and the United States.

Figs 5–7 show the conditional absolute errors (CAE) curves over time for BTF (blue curve) versus using the naive SIR model (red curve), ARIMA model (green curve) and generalized logistic growth curve (purple) projected forward from the same point in time. We define CAE as

$$\begin{array}{c} CAE=\{\begin{array}{cc} \text{AE} & ,\text{if}\;\text{the}\;\text{surge}\;\text{prediction}\;\text{is}\;\text{correct}\\ \infty & ,\text{if}\;\text{the}\;\text{surge}\;\text{prediction}\;\text{is}\;\text{wrong} \end{array} \end{array}$$

where AE is the absolute error.

Now the plots are zoomed in with the light blue shaded rectangular regions correspond to the time windows of the future surge of interest. Lower values of CAE indicate a better fit to the actually observed future data. Notice that if a surge is predicted incorrectly (i.e. a surge was not forecasted and it happened or vice versa), then the CAE is infinite.

In all 12 circumstances the BTF’s forecasts dominate naive model forecasts in terms of CAE. For Israel, the BTF CAE curve looks worse later in the shaded time window than earlier on; this corresponds to the projected peak for the surge being shifted too far to the right. For Australia, no important differences were found but this country was the negative control.

CAE curves, while useful, do not convey other important information regarding surge projections. For instance, it is of particular interest whether a surge projection accurately estimated peak height (within plus or minus 10 days) and/or location (within plus or minus 10 days). Table 1 breaks this down for our analysis. It indicates that for 5 countries we did indeed achieve peak height match, and for 8 countries we achieved peak location match. Contrast this to SIR, ARIMA and generalized logistic growth curve models which worked only for Australia, the negative control. Finally, we also compare BTF to the ECM method finding that BTF outperforms ECM. Although both methods borrow information from other countries, the constraints they impose on projections are different. The BTF makes use of SIR model which describes epidemic from a principled way, hence impose a strong constraint on the prediction result. The projection of BTF must satisfy the form of the solutions of SIR model, that is smoothness, unimodal etc. In a word, BTF has two constraints: the first is imposed implicitly by other counties when we borrow information from them, and the second is imposed by the epidemic dynamics. On the other hand, ECM only has the first constraint. So, we argue that is why BTF is preferred in the scenario of surge prediction.

**Table 1:** Countries where BTF projections matched surge peak height and location.

Peak Height Match	Peak Location Match
Israel	Germany
Australia	Australia
India*	India
Singapore	Singapore
UK	UK
	Japan
	South Africa
	US

Note: The * for India

under the peak height match column indicates the match is under a particular caveat described in the text.

We also found an interesting result regarding India’s projected surge. The second surge corresponded to the emergence of the delta variant of COVID, which produced a peak height of over 400K daily infections. Our projected estimate was only around 50K. However, it has also been estimated that at surge peak, fully 90% of the daily infection counts were attributable to the delta variant (https://clingen.igib.res.in/covid19genomes/). This means that 10% came from other existing variants found in other countries. Hence our projected peak approximates this number very accurately. We do not expect to project a peak for a new variant accurately using BTF, since the method, as currently formulated, cannot accommodate new variants.

## Justification for the matching

One of the features of a pandemic is that surges and recessions happen asynchronously across different countries. We are relying on the fact that finding a best matched country by time-shifting to create overlayed earlier surges will in fact provide useful information to coach future surge projections of interest. Thus it is necessary to say something regarding the optimality of this type of matching.

The correlation between asynchronous time series has been examined in what is termed lead-lag relationships between different financial markets [17]. For example, a link has been established between index futures and the cash market where the futures market tends to lead the cash market (see for instance [18]). The analysis of information flows between markets on short varying time intervals is an active area of research. In [17], the authors developed a method for estimating correlations from irregularly spaced transactions data.

For two stationary ARIMA processes, we can test for the presence of cross-correlation functions between the two asynchronous series. However, this approach is sensitive to the choice of lag length and cannot tell the directionality of causality, only the presence or absence of it. In addition, the statistic lacks power, as compared to regression-based tests discussed next.

One more clear way forward is to conduct a direct test for Granger causality [19], by regressing each variable on lagged values of itself and the other. This can be written as

$$\begin{array}{c} Y_{t}=\eta_{0}+\sum_{j=1}^{max(S_{1})-t}\eta_{j}Y_{t-j}+\sum_{k=1}^{max\left(S_{1,m}\right)-t^{\prime }}\kappa_{k}Z_{t^{\prime }-k}+\epsilon_{t}, \end{array}$$

where *ϵ*_*t*_ is white noise and *t*′ = *t* − *l*_*m*_, with *l*_*m*_ the lag between *S*_1_ and *S*_1,*m*_. Then the Granger causality between the two asynchronous time series can be assessed by testing whether the *κ*_*k*_ = 0 or not, using an F-test based on comparing nested residual sum of squares.

### Alternative strategy for matching using data enriched ARIMA models with lasso penalization

Assume the daily infection counts for country *A* in the currently ending surge are in time period *S*_1_. Assume a candidate country’s (*B*_*m*_) daily infection counts during a previous surge earlier than country *A*’s currently ending surge are in time period *S*_2_.

Assume for *A*, that *Y*_*t*_ follows the ARIMA(*p*, *d*, *q*) model Eq (2) with *t* ∈ *S*_1_; and country *B*_*m*_ also follows Eq (2), with *t* ∈ *S*_2_, but with autoregressive parameters (*α*_1_ + *ω*_*m*,1_), …, (*α*_*p*_ + *ω*_*m*,*p*_) and moving average parameters (*θ*_1_ + *ν*_*m*,1_), …, (*θ*_*q*_ + *ν*_*m*,*q*_) (assuming *p*, *d* and *q* are the same for both).

Then the two can be pooled via shrinkage and weighting as in [20] as the solution to the penalized joint log likelihood,

$$\begin{array}{c} l\left(A\right)+l\left(B_{m}\right)-\tau_{1}P\left(\omega_{m}\right)-\tau_{2}P\left(\nu_{m}\right), \end{array}$$

for penalty functions *P*(*ω*_*m*_) and *P*(*ν*_*m*_) and shrinkage parameters *τ*_1_ and *τ*_2_. Setting *P*(*ω*_*m*_) = ||*ω*_*m*_||_1_ and *P*(*ν*_*m*_) = ||*ν*_*k*_||_1_ corresponds to joint lasso shrinkage [14]. The shrinkage parameters can be estimated as part of the penalized maximum likelihood estimation process.

Then the best matching country would be,

$$\begin{array}{c} {\tilde{B}}_{m}=\text{arg}\;{\text{min}}_{k}\left({∥{\hat{\omega }}_{k}∥}_{1}+{∥{\hat{\nu }}_{k}∥}_{1}\right). \end{array}$$ (4)

## Connection to response coaching

For country *A*, let’s assume that *S*_1_ defines the time period of the first surge, and *S*_2_ the time period for the second surge. Let *Y*_*t*_ be the set of responses for country of interest *A*, *Z*_*tm*_ be the corresponding set of responses for country ${\tilde{B}}_{m}$, and *S*_1,*m*_ and *S*_2,*m*_ define the time periods for ${\tilde{B}}_{m}$’s first and second surge. Note that *S*_1_ > *S*_1,*m*_ and *S*_2_ > *S*_2,*m*_ by definition.

Then let $f_{\delta_{2}}\left(Y_{t}|t\in S_{2}\right)$ be the fit for *A* in *S*_2_, indexed by parameter vector *δ*_2_; and $f_{\delta_{2,m}}\left(Z_{tm}|t\in S_{2,m}\right)$ be the fit for ${\tilde{B}}_{m}$ in that country’s *S*_2,*m*_ indexed by parameter vector *δ*_2,*m*_. Also let *h*(*t*) be a function that maps from *S*_2,*m*_ to *S*_2_. These fits can be estimated using an SIR model. Remember that the interval corresponding to *S*_2,*m*_ is lagged with respect to the interval corresponding to *S*_2_.

Inspired by the response coaching idea of Tibshirani R and Hinton G. [21], we can write

$$\begin{array}{c} f_{\delta_{2,m}}\left(Y_{t},Z_{tm}|t\in S_{2}\right)=f_{\delta_{2,m}}\left(Y_{t}|t\in S_{2}\right)f_{\delta_{2,m}}(Z_{tm}|h^{-1}\left(t\right)\in S_{2,m}), \end{array}$$

where *δ*_2,*m*_ is a coaching parameter vector specific to *S*_2,*m*_ and shared with *A* during its *S*_2_. Thus the prediction of *Y*_*t*_ for *t* ∈ *S*_2_ can be coached by country ${\tilde{B}}_{m}$ via the shared parameter vector *δ*_2,*m*_ and estimated by

$$\begin{array}{c} \hat{f}\left(Y_{t}|t\in S_{2}\right)=f_{{\hat{\delta }}_{2,m}}\left(Y_{t}|t\in S_{2}\right), \end{array}$$

where ${\hat{\delta }}_{2,m}$ is estimated from the fit of ${\tilde{B}}_{m}$ in *t* ∈ *S*_2,*m*_ *and using the population characteristics of A* during *S*_2_.

The fact that *S*_1_ and *S*_1,*m*_ are not the same, and that *S*_2_ and *S*_2,*m*_ are not the same, but that we are expecting ${\tilde{B}}_{m}$ to be informative regarding *S*_2_ implies a periodic property of the pandemic across matched countries in time. That is, ${\tilde{B}}_{m}$ represents a country that experienced a very similar first surge and thus there is information to be gleaned about predicting *Y* by learning from ${\tilde{B}}_{m}$’s experience during their (earlier) second surge.

## Discussion

Making surge projections before the next surge begins is frankly necessary, given the highly infectious nature of many of the COVID-19 variants. Waiting until after an inflection point will simply mean that one is always playing catch up against the virus.

Our methodology attempts to do exactly this by employing a matching scheme to other candidate countries and then appealing to Granger causality in order to borrow from that matched country’s observed ensuing daily case counts. As we have discussed, once matching has occurred, the BTF projections themselves use a form of response coaching which can reduce variance over a non-coached model [21].

It should be emphasized that BTF projections cannot work well when a new variant emerges for the first time and it is the driver of a new surge. There is simply no hope to borrow strength from other countries. This is the reason our projections for India were not accurate due to the first-time emergence of the COVID-19 delta variant in early 2021. However, in the case of India, as explained in the performance assessment section of this paper, once the model was adjusted for the cases not pertaining to the delta variant, our projection did very well.

So how can one know whether the BTF technology could be of use in a prospective sense? One answer may lie in the recent work of Schioler H *et al.* [22], who developed a probabilistic model based on a hidden Markov model for infection spread and an approximation of a two stage sampling scheme to infer the probability of extinction of a current variant. Should this probability be low, then BTF may be useful in projecting future surges. Additional research is needed to adapt the methodology to allow for the possible emergence of new variants.

The CDC in the US released a community levels classification scheme designed to guide public health decision making regarding prevention strategies. It combines metrics about hospital admissions, bed usage due to COVID-19 cases and whether the new case rate in the last seven days has exceeded 200 per 100,000 people. The emphasis on early detection is clear, and that is why the CDC included syndromic surveillance in their metrics. Wastewater surveillance holds promise but because that surveillance does not provide broad coverage, it was not included in the list of metrics. To validate their choice for early warning indicators, the CDC assessed performance against community transmission indicators in predicting outcomes three weeks later and found that their early warning metrics produced higher predictive accuracy (as measured by area under the ROC curve) than the community level transmissions ([23]).

Taken in this context, the strength our methodology is the ability to make surge projections with good accuracy even further backwards in time. This could prove an important tool to accompany other early warning indicators. The weakness in application may arise from the fact that a suitably good country (or other region) match may not be found or that as explained above, a new variant emergency cannot be predicted.
